# The impact of mindfulness training and physical exercise on student academic burnout and academic achievement: a systematic review and meta-analysis

**DOI:** 10.3389/fpsyg.2026.1840246

**Published:** 2026-06-16

**Authors:** Wenxuan Wang, Ping Zhang

**Affiliations:** School of Physical Education, Qiqihar University, Qiqihar, Heilongjiang, China

**Keywords:** academic achievement, academic burnout, mental health, physical exercise, students

## Abstract

**Background:**

Although the benefits of physical exercise for students’ physical and mental health have been widely recognized, studies that systematically investigate the overall effects of physical exercise on students’ academic burnout and academic achievement remain insufficient. Most existing research focuses on a single dimension, lacking systematic integration and quantitative synthesis.

**Objective:**

This systematic review and meta-analysis (PROSPERO registration: CRD420261292068) aims to comprehensively evaluate the effects of physical exercise on various indicators of students’ academic burnout and academic achievement.

**Methods:**

We searched three databases: PubMed, Web of Science, and CNKI. Peer-reviewed Chinese and English randomized controlled trials published up to January 29, 2026 were included. Study quality was assessed using the Cochrane Risk of Bias tool.

**Results:**

Of the initially identified 12,086 studies, 35 met the inclusion criteria. Meta-analysis showed that Physical exercise significantly reduced students’ overall academic burnout (SMD = −0.45, 95% CI [−0.83, −0.07], *p* = 0.02) and was associated with a statistically significant but very small (trivial) improvement in overall academic achievement (SMD = 0.17, 95% CI [0.01, 0.34], *p* = 0.04). Physical exercise also showed statistically significant but very small beneficial effects on students’ spelling performance (SMD = 0.13, 95% CI [0.02, 0.24], *p* = 0.02), while descriptive analysis indicated favorable effects on mathematics performance, but had no significant effects on emotional burnout, cynicism, exhaustion, professional efficacy, reading, language skills, or cognitive inhibition. Further subgroup analysis revealed that physical exercise mainly promoted overall academic achievement and mathematics performance in primary school students.

**Conclusion:**

Physical exercise may significantly alleviate academic burnout and yield small but statistically significant improvements in academic achievement among students, especially overall academic achievement, mathematics performance and spelling performance in primary school students. These findings provide important evidence for promoting physical exercise among students to support their academic development and mental health.

**Systematic review registration:**

Identifier: CRD420261292068.

## 1 Introduction

Academic burnout refers to a state of negative psychological functioning and behavior characterized by declining interest, motivation, and engagement with learning, typically resulting from prolonged academic pressure (Kewei et al., 2019; Xin et al., 2017). Its core dimensions include emotional burnout, academic alienation, cynicism, among others (Kewei et al., 2019). In contrast to academic burnout, academic achievement is generally defined as the extent to which teachers, students, or educational institutions achieve educational objectives, typically measured through examinations or continuous assessments (Donnelly et al., 2017). Key influencing factors of academic achievement include individual cognitive ability (De Bruijn et al., 2020; Ernst et al., 2025; Tomporowski et al., 2007; Tomporowski et al., 2015) and school physical education curricula (Sallis et al., 1999) among others. As physical exercise constitutes a core element of school physical education, its potential impact on the academic achievement of students has not yet been systematically clarified. Physical exercise plays a positive role in the development of children and adolescents. It not only promotes their physical and mental health (Pinto-Escalona et al., 2024) and cognitive development (Duncan and Johnson, 2013; Layne et al., 2020; Melero-Cañas et al., 2021), but also is closely related to academic performance. Relevant studies have confirmed a positive correlation among higher physical activity, motor competence, physical fitness, and better academic performance (Lima et al., 2020). However, the specific manifestations of this association across the two dimensions of academic burnout and academic achievement remains controversial.

Existing randomized controlled trials (RCTs) investigating the effects of physical exercise on academic burnout and academic achievement among students have yielded inconsistent conclusions: Regarding academic burnout, some studies found that physical exercise can reduce overall academic burnout (Fisher et al., 2016; Kewei et al., 2019; Oró et al., 2021; Xin et al., 2017), whereas others reported no significant effect (van den Berg et al., 2019). For academic achievement, several studies verified that physical exercise can improve overall academic performance, mathematics performance, and spelling ability (Ahamed et al., 2007; Ardoy et al., 2013; Beck et al., 2016; De Bruijn et al., 2020; Donnelly et al., 2017), while others found no significant benefits for academic outcomes (van den Berg et al., 2019). Given these inconsistent findings, current systematic reviews still have several limitations: First, they have not conducted a detailed analysis of the effects of physical exercise on specific sub-indicators of academic burnout and academic achievement; Second, they have not explicitly focused on physical exercise as the intervention; some studies adopted psychological interventions instead; Third, most systematic reviews have included non-randomized studies, a practice that significantly weakens the causal interpretability of their conclusions. Furthermore, existing RCTs examining the impact of physical exercise on students’ academic achievement have used diverse intervention modes [e.g., acute cycling (Duncan and Johnson, 2013), aerobic training (Mavilidi et al., 2020)] and varying intervention durations [e.g., 30 min (Mavilidi and Vazou, 2021), one hour (Parmenter et al., 2019)], leading to markedly different outcomes. Inconsistencies in sample size, intervention protocols, and measurement tools across RCTs further hinder the consistency of research conclusions.

To address the above controversies and deficiencies, this study contributes to resolving key debates in the field at the theoretical level: First, it addresses the prevailing concern that “increasing time spent on physical exercise takes up classroom learning time and thus impairs academic achievement”; Second, it answers the question of “whether more physical exercise is always better”, given that existing research has found that excessive exercise can induce burnout and even aggravate academic burnout (Lima et al., 2020). Accordingly, this study argues that physical exercise is not only a facilitator of academic achievement but also a protective factor against academic burnout. At the practical level, this study provides clear guidance for physical exercise interventions targeting academic burnout and academic achievement among students: Based on existing literature, this study is expected to further confirm that physical exercise can specifically improve mathematics and spelling performance (Donnelly et al., 2017), and significantly alleviate academic burnout (Xin et al., 2017). This integrated dual-perspective analysis enriches the theory of dynamic balance in adolescent academic development. By including only randomized controlled trials (RCTs), this study can synthesize evidence across multiple studies through meta-analysis to improve the generalizability of findings, compared with single RCTs; meanwhile, compared with meta-analyses that incorporate non-RCTs, it achieves a higher level of evidence and strengthens the causal interpretability of the conclusions.

The primary aim of this study is to investigate the student population aged 6–24 years, a broad but educationally meaningful range spanning childhood, adolescence, and early adulthood. This age spectrum was intentionally selected to capture the full continuum of academic pressure and developmental plasticity, allowing a comprehensive assessment of physical exercise effects on academic burnout (including its sub-dimensions) and academic achievement (including overall, mathematics, and spelling performance) across distinct educational stages. Using meta-analysis, this study further explores the mechanism underlying how physical exercise influences students’ academic development, and on the basis of verifying the direction of effects, we quantify the alleviative effect of physical exercise on academic burnout and its facilitative effect on academic achievement.

## 2 Methods

### 2.1 Literature search strategy

This systematic review and meta-analysis was conducted and reported in strict accordance with the Preferred Reporting Items for Systematic Reviews and Meta-Analyses (PRISMA) 2020 statement.

We comprehensively collected relevant randomized controlled trials (RCTs) that meet the inclusion criteria, ensure the comprehensiveness, systematicness, and reproducibility of the search, and avoid missing relevant literature, core databases at home and abroad were selected based on the research topic: China National Knowledge Infrastructure (CNKI); English databases (PubMed, Web of Science). The search period was from the establishment of each database to January 29, 2026. A combination of subject headings and free-text terms was employed to determine Chinese and English search terms separately, covering two core dimensions: intervention measures and primary outcomes, to minimize search bias. Full, reproducible search strings using standard Boolean operators (OR, AND) are provided below for transparency. English search string: (“Physical Exercise” OR “Exercise” OR “Physical Activity”) AND (“Academic Burnout” OR “Psychological Burnout” OR “Burnout” OR “Burnout Syndrome”) AND (“Academic Achievement” OR “Academic Success”). Chinese database search string: (“Physical Exercise” OR “Physical Activity” OR “Exercise”) AND (“Academic Burnout” OR “Student Burnout” OR “Psychological Burnout”) AND (“Academic Achievement” OR “Academic Performance”). Gray literature including conference abstracts, dissertations, preprints, and unpublished data was not searched or included. The detailed search strategy is shown in the appendix.

### 2.2 Eligibility criteria

#### 2.2.1 Inclusion criteria

(a) Study type: randomized controlled trial (RCT) design; (b) Study subjects: individual students aged 6–24 years without chronic diseases affecting physical activity participation (including primary school, middle school, university, and graduate students). This age range covers major developmental and educational stages from childhood to early adulthood, allowing comprehensive evaluation of exercise effects across distinct developmental periods; (c) Intervention measures: The experimental group received physical exercise, which was explicitly defined as structured, purposeful bodily movement performed to improve or maintain physical fitness, health, or motor function. This included aerobic exercise, sports training, physical activity games, and academically integrated physical activity. Mindfulness was included only when combined with physical exercise (mind–body integrated exercise); pure mindfulness without bodily movement was excluded. No restrictions were set on exercise type, duration, frequency, or intensity; (d) Control measures: the control group did not undergo any form of mindfulness training or physical exercise (no additional physical activity intervention) and only maintained a normal study and living rhythm; (e) Outcome indicators: including academic burnout or academic achievement. Academic burnout included specific indicators such as overall academic burnout, cynicism, emotional burnout, professional efficacy, and exhaustion. Academic achievement included specific indicators such as overall academic achievement, mathematics, spelling, reading, language, and cognitive inhibition; (f) Definition of Physical Exercise: For the purpose of this review, physical exercise was strictly defined as any structured, repetitive bodily movement that increases physical load and aims to enhance physical fitness, health, or motor ability. Mindfulness practices in the included studies were always integrated with physical exercise (e.g., mindful movement, yoga-based exercise) and thus classified as mind–body physical exercise rather than independent psychological interventions. This definition ensures conceptual clarity and consistency across included studies.

#### 2.2.2 Exclusion criteria

(a) Study type: non-RCT studies, including quasi-experimental studies and observational studies (cohort studies, case–control studies, etc.); (b) Study subjects: those who did not meet the above inclusion criteria, or those with severely missing baseline data of study subjects and unable to extract valid data; (c) Intervention measures: unclear intervention measures, unquantifiable dose/course of treatment, or unclear differences in intervention measures between the experimental group and the control group; (d) Outcome indicators: those that did not include the preset outcome indicators of this study, or those with severely missing outcome indicator data and unable to convert or extract; (e) Others: duplicate publications, for which only the most recent or largest study was retained, conference abstracts (those without complete data), dissertations (if journal papers have been published, journal papers were prioritized for inclusion), and literature with extremely low quality (e.g., contradictory data, internal inconsistencies) that could not be quality-assessed.

### 2.3 Literature screening and data extraction

#### 2.3.1 Literature screening

The double-independent screening method was adopted. Two trained researchers (WW and PZ) initially screened the retrieved literature titles and abstracts according to the preset inclusion and exclusion criteria to exclude literature that was obviously inconsistent with the criteria; for the literature that passed the initial screening, the full text was further obtained, and secondary screening was conducted after careful reading to determine the finally included literature. If the two researchers had inconsistent screening opinions, the dispute was resolved through discussion.

#### 2.3.2 Data extraction

Two researchers (WW and PZ) independently extracted key data from the included literature using a pre-established data extraction form. After data extraction was completed, cross-checking was performed to ensure accuracy. Any discrepancies were resolved through discussion. The extracted content should be comprehensive and standardized, mainly including: (a) Basic literature information: first author, year of publication, country/region, sample size (experimental group/control group); (b) Study design information: randomization method, blinding type (single-blind, double-blind, triple-blind, or no blinding), allocation concealment, follow-up time, loss to follow-up rate, and reasons for loss to follow-up; (c) Intervention and control information: intervention measures and control measures; (d) Outcome indicator information: measurement methods of each outcome indicator, outcome data of the experimental group and the control group; (e) Quality assessment-related information: quality control measures mentioned in the literature, specific implementation details of randomization methods, etc.

### 2.4 Quality assessment

An internationally recognized RCT quality assessment tool was used to conduct double-independent assessment of the methodological quality of the included literature. The assessors scored/rated strictly in accordance with the tool standards, cross-checked the assessment results, and resolved discrepancies through discussion or arbitration by a third researcher. Cochrane Risk of Bias Tool (RoB 1.0): applicable to all RCTs, it assesses the risk of bias from 7 core domains, including “bias arising from the randomization process, bias due to allocation concealment, bias due to blinding of participants and personnel, bias due to blinding of outcome assessment, bias due to incomplete outcome data, bias due to selective outcome reporting, and other biases”. Each domain is rated at one of three levels: “low risk of bias, high risk of bias, and unclear risk of bias”. Finally, the overall risk of bias of each literature was comprehensively judged.

### 2.5 Data synthesis and analysis

Data synthesis and analysis were performed using RevMan 5.4 and Stata 16.0. The version of the software used was clearly stated, all statistical analysis steps were preset in advance, and the analysis was strictly performed in accordance with meta-analysis standards.

#### 2.5.1 Heterogeneity test

First, heterogeneity test was performed on all included studies to judge the consistency of effect sizes among different studies and avoid deviations in synthesis results caused by excessive heterogeneity. (a) Test methods: *Q* test (*χ*^2^ test) and *I*^2^ statistic were used. The *Q* test was used to determine the existence of heterogeneity (*p* < 0.1 indicated statistical heterogeneity), and the *I*^2^ statistic was used to quantify the degree of heterogeneity, following the widely cited Higgins et al. thresholds: 0–25% = low heterogeneity, 25–50% = moderate heterogeneity, 50–75% = substantial heterogeneity, and >75% = considerable heterogeneity; (b) Heterogeneity handling: if there was moderate to high heterogeneity (*I*^2^ ≥ 50%), a random-effects model was used for data synthesis, and the source of heterogeneity was analyzed simultaneously (sensitivity analysis was used to explore the causes of heterogeneity); If there was high heterogeneity (*I*^2^ > 75%), we still proceeded with meta-analysis using a random-effects model, as the aim was to pool population-level effects across diverse study designs and intervention characteristics. Subgroup and meta-regression analyses were then conducted to explore potential sources of between-study variability, and the causes of heterogeneity and handling ideas were detailed in the discussion section.

#### 2.5.2 Effect size selection and data synthesis

An appropriate effect size was selected according to the type of outcome indicator: for continuous outcome indicators (such as overall academic burnout, emotional burnout, etc.), the standardized mean difference (SMD) was used as the effect size for continuous outcomes with inconsistent measurement units across studies, and 95% confidence interval (95% CI) was reported simultaneously. During effect size synthesis, a fixed-effects model (no/mild heterogeneity) or a random-effects model (moderate heterogeneity) was selected according to the results of the heterogeneity test. The effect size of each study and the pooled effect size were intuitively displayed through a forest plot, which should include the weight, effect size, 95% CI of each study, and the statistical test result (*p*-value) of the pooled effect size.

#### 2.5.3 Sensitivity analysis

It was used to test the stability and reliability of meta-analysis results and exclude the impact of abnormal studies on the pooled results. Common methods included: excluding each included literature one by one, re-conducting heterogeneity test and effect size synthesis, and observing whether the pooled effect size changed significantly.

#### 2.5.4 Publication bias detection

It was used to assess potential publication bias. Common methods included: (a) Funnel plot analysis was performed based on effect size and standard error, with symmetry assessed to judge potential publication bias; (b) Quantitative test: Egger’s test was adopted, *p* > 0.05 indicated no obvious publication bias, and *p* < 0.05 indicated the presence of publication bias. If there was publication bias, the possible reasons (e.g., unpublished negative studies with small sample sizes) should be explained in the discussion section, and its impact on the results should be analyzed.

## 3 Results

### 3.1 Study selection

**Figure 1:**
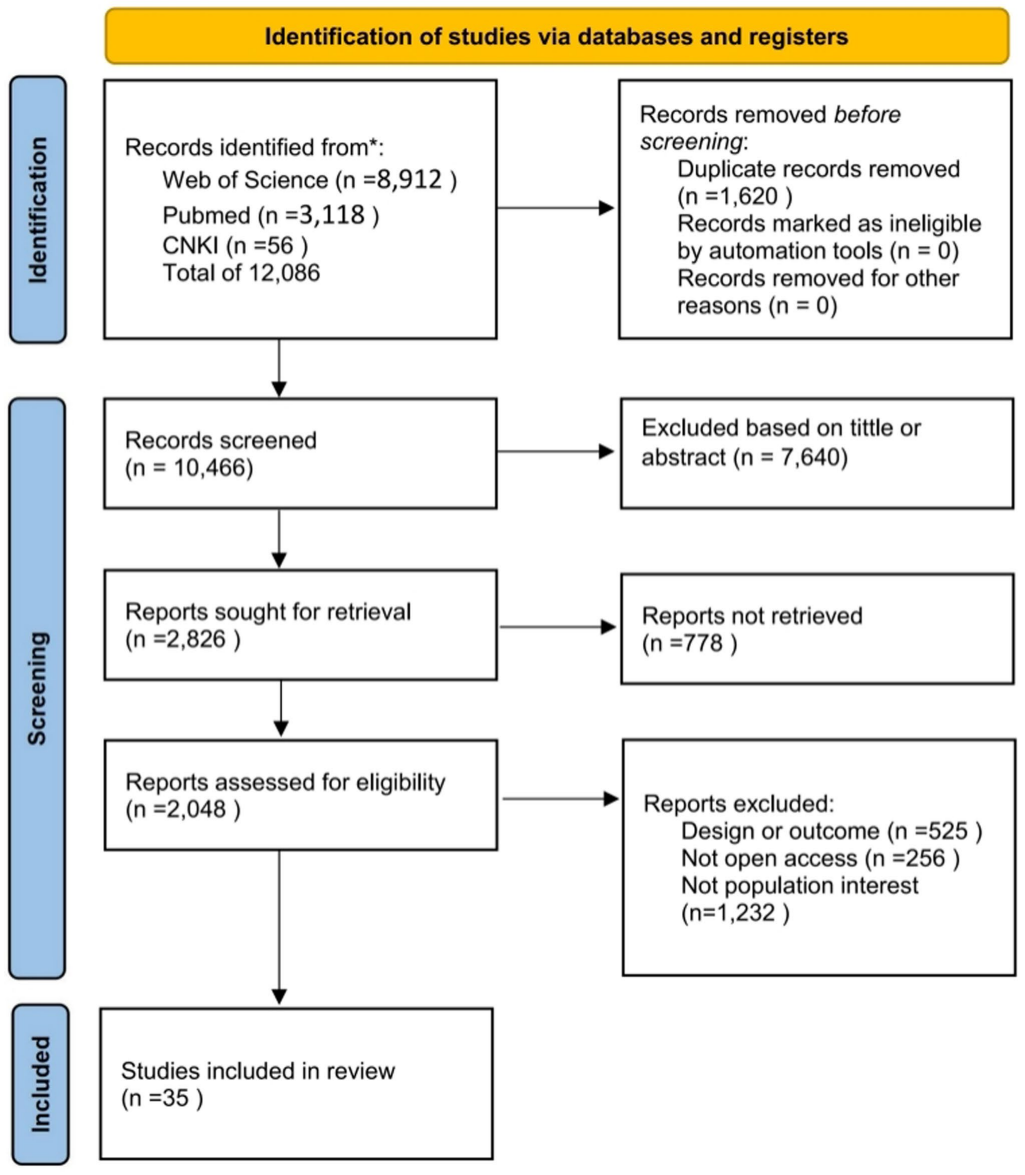
PRISMA 2020 flow diagram of study selection process for the systematic review and meta-analysis. The diagram illustrates the number of records identified, screened, assessed for eligibility, and ultimately included in the final quantitative synthesis.

A total of 12,086 studies were identified from the three searched databases. After removing 1,620 duplicate records, 10,466 records were screened, and 7,640 were excluded based on title and abstract. Then, 2,826 records were sought for retrieval; 778 reports were not retrieved, and 2,048 full-text articles were assessed for eligibility. After full-text evaluation, 2,013 articles were excluded. Finally, 35 articles met the eligibility criteria and were included in this systematic review and meta-analysis (Figure 1).

### 3.2 Characteristics of the included studies

Thirty-five RCTs were included in this meta-analysis, with a total sample size of 12,483 participants, including 7,154 in the intervention group and 5,329 in the control group. All included studies were published in Chinese or English, including 2 Chinese and 33 English articles published between 1999 and 2024. The literature search was conducted up to January 2026, yet no newly published studies after 2024 fulfilled the predefined inclusion criteria, covering regions including China, the United States, and Europe. The included participants covered a wide age range of 6–24 years and different regional populations, making the sample reasonably representative of children and adolescents worldwide.

The basic characteristics are summarized as follows: all participants were students aged 7.4 to 24 years, including primary, junior high, senior high, and university students. The gender and age distribution were comparable between the intervention and control groups across studies, indicating good homogeneity and reducing the influence of baseline differences on intervention effects.

For interventions, the experimental groups all received standardized physical exercise, including aerobic exercise (cycling, running), mind–body exercise (karate), and physical activity (school-based activities, active exercise games); mindfulness was treated as an independent non-exercise intervention. Among them, 7 studies used aerobic exercise, 6 used mindfulness, and 22 used physical activity. The intervention frequency ranged from 1 to 8 sessions per week, with single-session duration varying from 5 to 120 min, and intervention period from 2 weeks to 24 months. Most interventions were conducted in school playgrounds or gymnasiums, some guided by professional physical education teachers. Control groups received no additional physical exercise (only routine class teaching) or non-exercise interventions (e.g., general health education). Details are shown in Table 1. Notably, some included studies did not report complete data on age, exercise intensity, or intervention duration (marked as N/A). These missing data may limit the interpretability of subgroup and meta-regression analyses and represent a minor limitation of this study.

**Table 1:** Characteristics of enrolled studies.

Order number	Inclusion criteria	Area	Participant sample (*n*)	Sex (male, *n*, %)	Age (*M* ± SD)	Crowd	Intervention method	Intensity	Intervention time	Survey tools	Time of duration (week)	Outcome
1	Xin et al. (2017)	China	IG: 30	IG: 12 (40%)	19~22 (range)	College student	IG: mindfulness	N/A	30 min per session, 7 times per week	MBI-SS	2	Overall academic burnout
	RCT		CG: 30	CG: 11 (37%)			CG: no exercise intervention					
2	Kewei et al. (2019)	China	IG: 12	IG: 6 (50%)	N/A	College student	IG: mindfulness	N/A	N/A	MBI-SS	8	Overall academic burnout
	RCT		CG: 19	CG: 6 (31.6%)			CG: no exercise intervention			MAAS		
3	O’Driscoll et al. (2019)	Ireland	IG: 51	IG: 17 (33.3%)	IG: 33 ± 64.7	College student	IG: mindfulness	N/A	Every 2 h/4 times per week	MBI-SS	4	Occupational
												Efficiency
												Exhaust
	RCT		CG: 48	CG: 16 (33.3%)	CG: 29 ± 20.4		CG: no exercise intervention					Be cynical
4	Oró et al. (2021)	Spain	IG: 68	N/A	20.28 ± 1.54	College student	IG: mindfulness	N/A	Every 2 h/a total of 8 times	MBI-SS	16	Overall academic burnout
												Emotional burnout
	RCT		CG: 75				CG: no exercise intervention					Be cynical
5	Clarkson et al. (2019)	United Kingdom	IG: 8	N/A	N/A	College student	IG: mindfulness	N/A	Every 2 h twice weekly	MBI-SS	8	Exhaust
												Occupational efficiency
	RCT		CG: 6				CG: no exercise intervention					Be cynical
6	de Vibe et al. (2013)	Norway	IG: 144	IG: 26 (18%)	IG: 23.6 ± 4.7	College student	IG: mindfulness	N/A	2.5 h per session, 8 times per week	MBI-SS	7	Overall academic burnout
	RCT		CG: 144	CG: 43 (30%)	CG: 24.0 ± 5.7		CG: no exercise intervention					
7	Fisher et al. (2016)	Netherlands	IG: 40	IG: 9 (18%)	IG: 20.9 ± 2.5	College student	IG: aerobic exercise	Low-intensity	Once every hour, twice weekly	MBI-SS	6	Emotional burnout
										UBOS-S		Overall academic burnout
	RCT		CG: 48	CG: 10 (20.4%)	CG: 20.7 ± 2.2		CG: no exercise intervention			FAS		Demand recovery
8	Ahamed et al. (2007)	Canada	IG: 214	N/A	IG: 10.2 ± 0.6	Primary school	IG: physical exercise	Moderate to severe	150 min per session, once per week	CAT-3	64	Overall academic achievement
	RCT		CG: 73		CG: 10.2 ± 0.6		CG: no exercise intervention					
9	Ardoy et al. (2013)	Spain	IG: 49	N/A	IG: 13 ± 0.1	Junior middle school student	IG: physical exercise	High strength	55 min per session, twice weekly	N/A	16	Language
	RCT		CG: 18		CG: 13.8 ± 0.1		CG: no exercise intervention					Maths
10	Beck et al. (2016)	Denmark	IG: 165	N/A	IG: 7.5 ± 0.03	Primary school	IG: physical exercise	N/A	Once every hour, three times per week	SPPDT	6	Maths
	RCT		CG: 149		CG: 7.5 ± 0.02		CG: no exercise intervention					
11	De Bruijn et al. (2020)	Netherlands	IG: 461	IG: 221 (48%)	IG: 9.33 ± 0.64	Primary school	IG: physical exercise	Moderate to severe	0.5 h per session, twice weekly	N/A	14	Read
												Maths
	RCT		CG: 430	CG: 219 (51%)	CG: 9.15 ± 0.67		CG: no exercise intervention					Spell
12	Donnelly et al. (2017)	United States	IG: 9	IG: 164 (51.9%)	IG: 8.1 ± 0.6	Primary school	IG: physical exercise	Moderate to severe	55 min per session, once per week	SOFIT	36	Read
												Maths
	RCT		CG: 8	CG: 145 (45.9%)	CG: 8.1 ± 0.6		CG: no exercise intervention			WIAT-III		Spell
13	Lima et al. (2020)	Austria	IG: 1012	IG: 441 (43.58%)	IG: 14.99 ± 1.04	Senior high school student	IG: physical exercise	N/A	Once every hour, four times per week	N/A	24	Overall academic achievement
	RCT		CG: 188	CG: 22 (39.86%)	CG: 15.15 ± 1.02		CG: no exercise intervention					
14	Mavilidi and Vazou (2021)	Australia	IG: 355	IG: 169 (47.60%)	9~11 (range)	Primary school	IG: physical exercise	N/A	0.5 h per session, 7 times per week	N/A	8	Maths
	RCT		CG: 205	CG: 98 (47.80%)			CG: conventional teaching					
15	Melero-Cañas et al. (2021)	Spain	IG: 113	N/A	14.63 ± 1.38	Junior middle school student	IG: sports games	N/A	N/A	N/A	36	Language
												Maths
	RCT		CG: 37				CG: no exercise intervention					Cognitive inhibition
16	Mullender-Wijnsma et al. (2016)	Netherlands	IG: 113	IG: 116 (23%)	IG: 8.0 ± 0.72	Primary school	IG: physical exercise	N/A	Three times a week	N/A	44	Read
												Maths
	RCT		CG: 37	CG: 110 (22%)	CG: 8.2 ± 0.74		CG: conventional teaching					Spell
17	Solberg et al., 2021a,b	Norway	IG: 20	N/A	IG: 14.9 ± 0.3	Junior middle school student	IG: aerobic physical exercise	Moderate intensity	Once every hour/once per week	N/A	36	Maths
	RCT		CG: 10		CG: 14.9 ± 0.3		CG: no exercise intervention					Read
18	Pinto-Escalona et al. (2024)	Spain	IG: 388	IG: 381 (52.9%)	IG: 7.4 ± 0.5	Primary school	IG: karate	N/A	2 h per week	N/A	48	Overall academic achievement
	RCT		CG: 333	CG: 373 (51.8%)	CG: 7.4 ± 0.4		CG: no exercise intervention					
19	Parmenter et al. (2019)	Switzerland	IG: 142	IG: 21 (14.78%)	IG: 7.96 ± 0.36	Primary school	IG: physical exercise	N/A	Once every hour, 5 times per week	N/A	20	Read
												Maths
	RCT		CG: 142	CG: 22 (15.49%)	CG: 7.82 ± 0.41		CG: no exercise intervention					Spell
20	Centeio et al. (2021)	Honolulu	IG: 377	IG: 289 (46%)	IG: 9.92 ± 0.36	Primary school	IG: physical exercise	N/A	N/A	N/A	30	Maths
	RCT		CG: 251	CG: 320 (51%)	CG: 10.02 ± 0.37		CG: no exercise intervention					Read
21	Mavilidi et al. (2019)	Australia	IG: 58	IG: 53 (60.9%)	IG: 9.11 ± 0.62	Primary school	IG: physical exercise	N/A	5 min per session, 5 times per week	N/A	4	Maths
	RCT		CG: 29	CG: 20 (69%)	CG: 9.2 ± 0.56		CG: no exercise intervention					
22	van Wouwe et al. (2018)	Switzerland	IG: 265	IG: 122 (46%)	IG: 9.22 ± 0.76	Primary school	IG: physical exercise	N/A	N/A	N/A	20	Overall academic achievement
	RCT		CG: 398	CG: 203 (51%)	CG: 9.28 ± 0.66		CG: no exercise intervention					
23	Wang et al. (2023)	China	IG: 1012	IG: 511 (50.5%)	IG: 9.21 ± 0.62	Primary school	IG: physical exercise	N/A	N/A	N/A	48	Maths
	RCT		CG: 1020	CG: 481 (47.2%)	CG: 9.23 ± 0.62		CG: no exercise intervention					
24	Lubans et al. (2018)	Australia	IG: 693	IG: 430 (59%)	IG: 12.96 ± 0.56	Junior middle school student	IG: physical exercise	Moderate to severe	N/A	N/A	16	Maths
	RCT		CG: 728	CG: 360 (51.9%)	CG: 12.9 ± 0.52		CG: no exercise intervention					
25	Sallis et al. (1999)	United States	IG: 754	IG: 393 (52.2%)	IG: 9.5 ± 0.43	Primary school	IG: physical exercise	N/A	30 min per session, 3 times per week	MAT6 and MAT7	72	Maths
	RCT		CG: 387	CG: 203 (52.6%)	CG: 9.6 ± 0.52		CG: no exercise intervention					Read
26	Layne et al. (2020)	United States	IG: 19	IG: 14 (73.7%)	8~9 (range)	Primary school	IG: physical exercise	N/A	N/A	N/A	4	Maths
	RCT		CG: 21	CG: 17 (80.9%)			CG: no exercise intervention					Cognitive inhibition
27	Mavilidi et al. (2020)	Australia	IG: 66	N/A	11~12 (range)	Primary school	IG: aerobic exercise	High strength	10 min per session, twice weekly	N/A	10	Maths
	RCT		CG: 35				CG: no exercise intervention					
28	van den Berg et al. (2019)	Netherlands	IG: 170	IG: 85 (50%)	IG: 11.0 ± 0.42	Primary school	IG: physical exercise	Moderate to severe	5–8 min per session, 4 times per week	N/A	5	Cognitive inhibition
	RCT		CG: 153	CG: 77 (45%)	CG: 10.9 ± 0.48		CG: no exercise intervention					
29	Duncan and Johnson (2013)	United Kingdom	IG: 18	IG: 9 (50%)	IG: 9.8 ± 1.4	Primary school	IG: aerobic exercise	Moderate to severe	20 min each time	WRAT4	Acute	Read
												Maths
	RCT		CG: 18	CG: 9 (50%)	CG: 9.8 ± 1.4		CG: no exercise intervention					Spell
												Language
30	Fiorilli et al. (2021)	Italy	IG: 91	IG: 40 (44%)	IG: 9.58 ± 0.85	Primary school	IG: physical exercise	Moderate to severe	15 min per session, 3 times per week	SCWT	Acute	Maths
	RCT		CG: 50	CG: 28 (56%)	CG: 9.66 ± 0.82		CG: no exercise intervention					
31	Harveson et al. (2019)	United States	IG: 63	IG: 57 (90.5%)	13.7 ± 0.47	Junior middle school student	IG: aerobic exercise and resistance training	N/A	20 min per session, once per week	N/A	3	Maths
	RCT		CG: 63	CG: 57 (90.5%)			CG: no exercise intervention					
32	Hillman et al. (2009)	United States	IG: 20	IG: 12 (60%)	IG: 9.6 ± 0.7	Primary school	IG: aerobic exercise	Moderate	N/A	N/A	Acute	Read
												Maths
	RCT		CG: 20	CG: 12 (60%)	CG: 9.6 ± 0.7		CG: no exercise intervention					Spell
33	Howie et al. (2015)	United States	IG: 94	IG: 34 (36.2%)	IG: 10.7 ± 0.6	Primary school	IG: aerobic exercise	Moderate to severe	Each session lasts 5–20 min.	N/A	Acute	Maths
	RCT		CG: 94	CG: 34 (36.2%)	CG: 10.7 ± 0.6		CG: no exercise intervention					
34	Kawabata et al. (2021)	Singapore	IG: 40	IG: 10 (25%)	IG: 16.1 ± 0.9	Senior high school student	IG: physical exercise	N/A	20 min per session, 5 times per week	WIAT-III	Acute	Maths
	RCT		CG: 42	CG: 8 (19.1%)	CG: 15.9 ± 1.2		CG: no exercise intervention					
35	Pontifex et al. (2013)	United States	IG: 20	IG: 14 (70%)	IG: 9.3 ± 0.3	Primary school	IG: aerobic exercise	Moderate	20 min each time	WRAT3	Acute	Read
												Maths
	RCT		CG: 20	CG: 14 (70%)	CG: 9.8 ± 0.1		CG: no exercise intervention					Spell

MBI-SS, Maslach Burnout Inventory-Student Survey; BMS, Burnout Measure Short; UBOS-S, Student Burnout Scale and Emotional Burnout Scale; FAS, Burnout Assessment Scale; CAT-3, Standardization Achievement Test; Standardized Paper-and-Pencil Diagnostic Test (standardized paper-and-pencil diagnostic test); SOFIT, System for Observing Fitness Instruction Time; SPPDT, Standardized paper-and-pencil diagnostic test; WIAT-III, Wechsler Individual Achievement Test-Third Edition; MAT6andMAT7, Metropolitan Achievement Tests; WRAT4, Wide Ranging Achievement Task; MAAS, Mindful Attention Awareness Scale; SCWT, Stroop Color and Word Test; WRAT3, Wide Range Achievement Test, 3rd Edition; N/A, Not available; relevant data were not reported in the original studies.

For outcome measures, all studies reported the core outcomes predefined in this meta-analysis. Seven studies reported academic burnout measured by the Maslach Burnout Inventory-Student Survey (MBI-SS), including overall burnout, emotional burnout, cynicism, exhaustion, and professional efficacy. Twenty-eight studies reported academic achievement assessed by final exam scores, standardized academic tests, or average subject scores, including mathematics, spelling, reading, language, and cognitive inhibition. All outcomes had clear criteria and complete extractable data.

### 3.3 Risk of bias assessment

**Figure 2:**
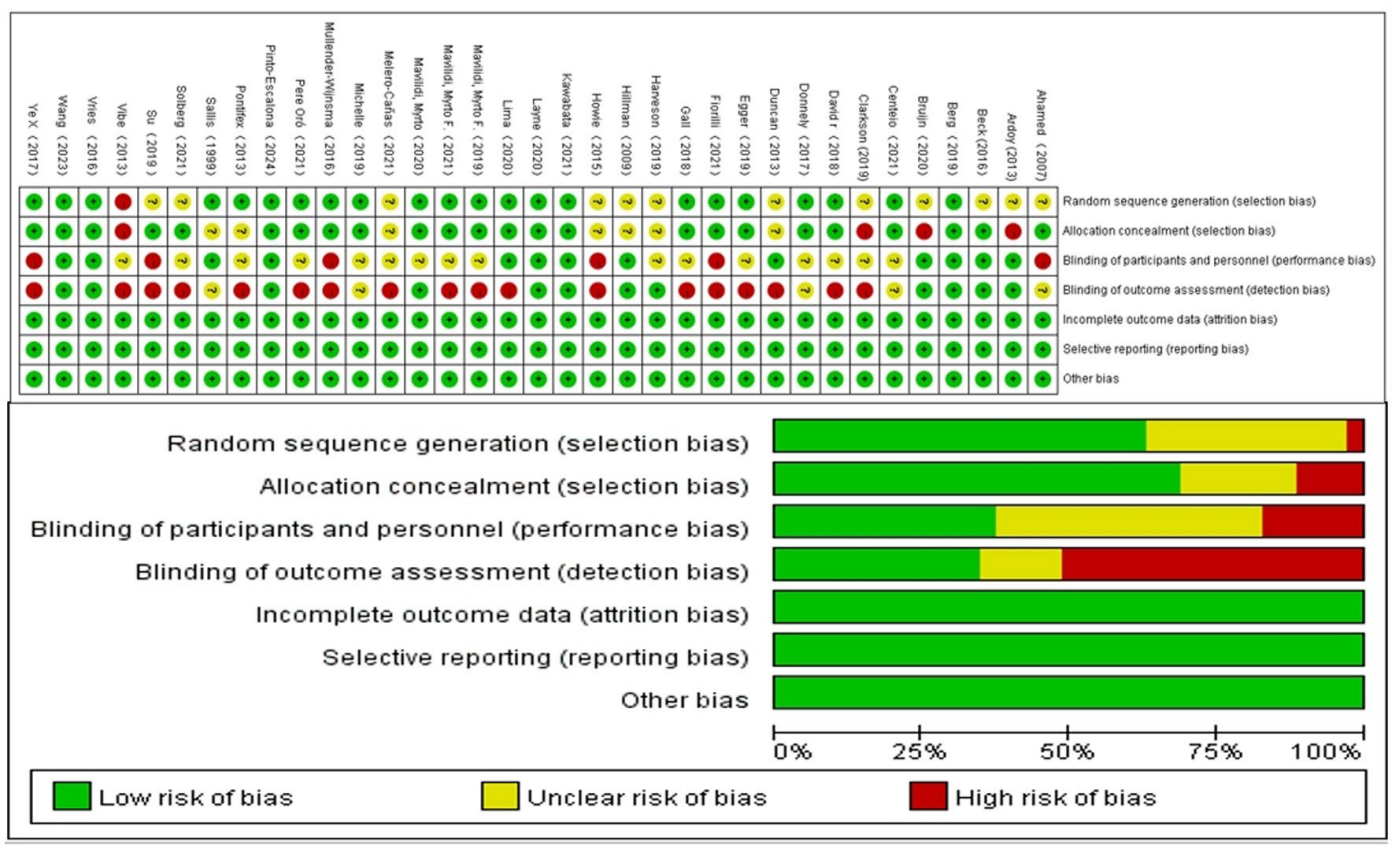
Risk of bias summary for the 35 included randomized controlled trials, assessed using the Cochrane Risk of Bias Tool (RoB 1.0). Each domain is rated as low risk, unclear risk, or high risk of bias.

Risk of bias is summarized in Figure 2. Overall, the risk of bias among the 35 included trials was acceptable. Adequate random sequence generation was reported in 20 trials (57%), adequate allocation concealment in 23 trials (66%), blinding of participants and personnel in 15 trials (43%), and blinding of outcome assessors in 14 trials (40%). No incomplete outcome data or selective outcome reporting was observed in 35 trials (100%), indicating low attrition bias and reporting bias (see Table 2).

**Table 2:** Bias analysis.

Outcome	Egger	
	*t*	*p*
Overall academic performance	0.80	0.442
Math scores	0.82	0.418

### 3.4 Results of meta-analysis

#### 3.4.1 Effects of mindfulness training and physical exercise on student academic burnout

##### 3.4.1.1 Effect of mindfulness training and physical exercise on overall academic burnout

Five studies (de Vibe et al., 2013; Fisher et al., 2016; Kewei et al., 2019; Oró et al., 2021; Xin et al., 2017) with 610 participants were included. Heterogeneity test showed *I*^2^ = 62%, *p* = 0.03, indicating moderate heterogeneity; thus, a random-effects model was used. The pooled effect showed that overall burnout was significantly lower in the exercise group (SMD = −0.33, 95% CI [−0.63, −0.04], *p* = 0.03). Mindfulness training and physical exercise significantly reduced overall burnout among students.

##### 3.4.1.2 Effect of mindfulness training and physical exercise on emotional burnout

Two studies (Fisher et al., 2016; Oró et al., 2021) with 231 participants were included. Heterogeneity test showed *I*^2^ = 3%, *p* = 0.31; a fixed-effects model was used. No significant difference was found between groups (SMD = −0.05, 95% CI [−0.31, 0.21], *p* = 0.71). Mindfulness training and physical exercise had no significant effect on emotional burnout.

##### 3.4.1.3 Effect of mindfulness training and physical exercise on cynicism

Three studies (Clarkson et al., 2019; O’Driscoll et al., 2019; Oró et al., 2021) with 256 participants were included. Heterogeneity test showed *I*^2^ = 0%, *p* = 0.41; a fixed-effects model was used. No significant difference was found (SMD = −0.20, 95% CI [−0.45, 0.05], *p* = 0.11). Mindfulness training and physical exercise had no significant effect on cynicism.

##### 3.4.1.4 Effect of mindfulness training and physical exercise on exhaustion

Two studies (Clarkson et al., 2019; O’Driscoll et al., 2019) with 113 participants were included. Heterogeneity test showed *I*^2^ = 0%, *p* = 0.51; a fixed-effects model was used. No significant difference was found (SMD = −0.05, 95% CI [−0.42, 0.32], *p* = 0.79). Mindfulness training and physical exercise had no significant effect on exhaustion.

##### 3.4.1.5 Effect of mindfulness training and physical exercise on professional efficacy

**Figure 3:**
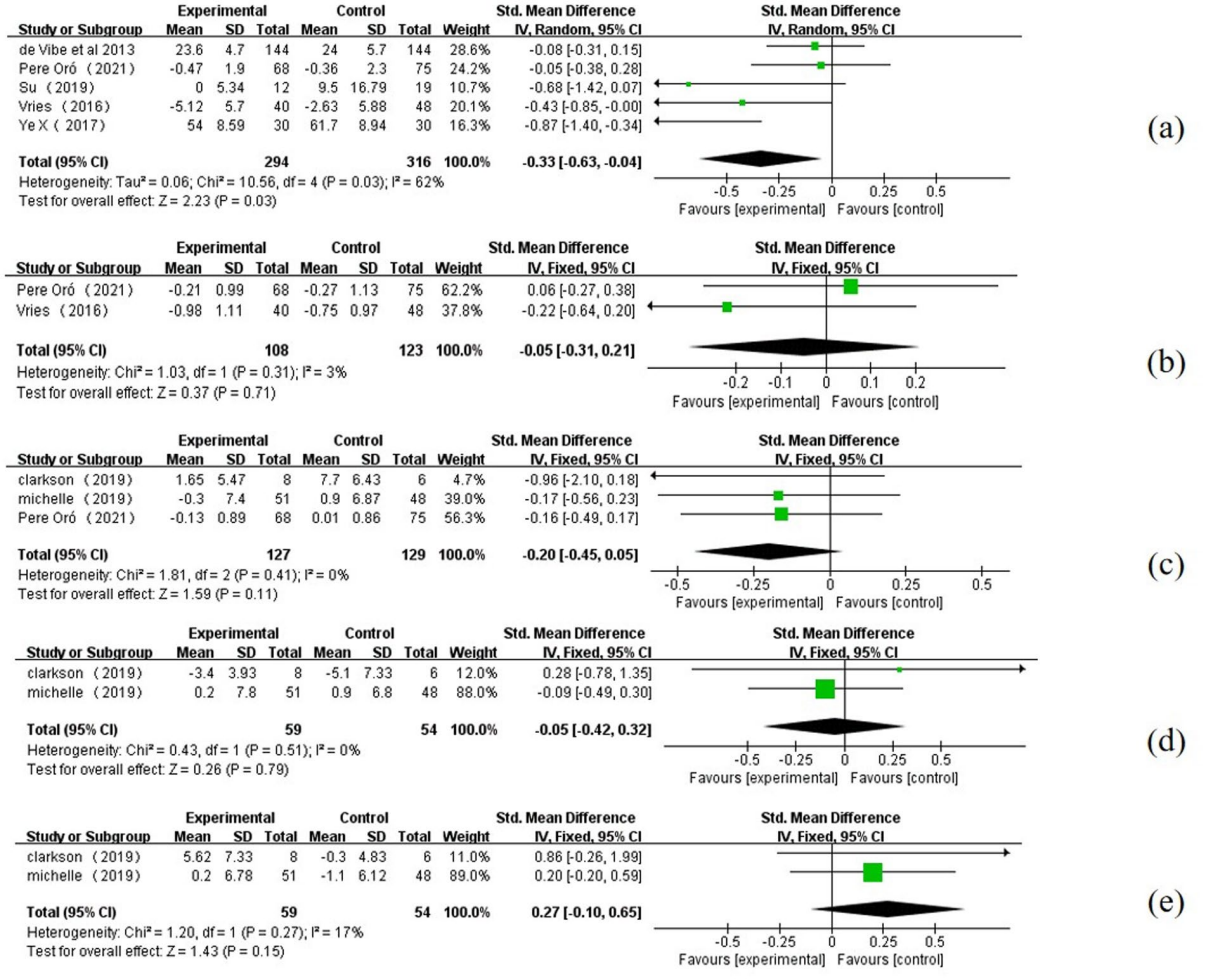
Forest plot illustrating the impact of mindfulness training and physical exercise on student academic burnout. **(a)** Overall academic burnout **(b)** Emotional burnout **(c)** Cynicism **(d)** Exhaustion **(e)** Occupational efficacy.

Two studies (Clarkson et al., 2019; O’Driscoll et al., 2019) with 113 participants were included. Heterogeneity test showed *I*^2^ = 17%, *p* = 0.27; a fixed-effects model was used. No significant difference was found (SMD = 0.27, 95% CI [−0.10, 0.65], *p* = 0.15). Mindfulness training and physical exercise had no significant effect on professional efficacy (see Figure 3).

#### 3.4.2 Effects of physical exercise on students’ academic achievement

##### 3.4.2.1 Effect on overall academic achievement

**Figure 4:**
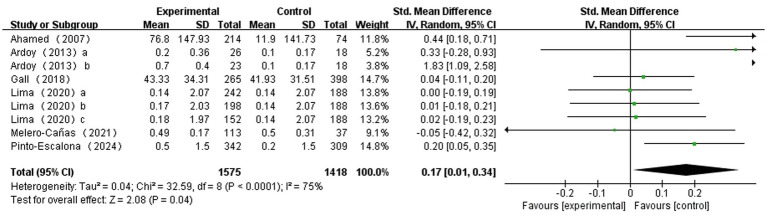
Forest plot of the effect of physical exercise on students’ overall academic achievement. The pooled standardized mean difference (SMD) and 95% confidence interval (CI) are presented.

Six studies (Ahamed et al., 2007; Ardoy et al., 2013; Lima et al., 2020; Melero-Cañas et al., 2021; Pinto-Escalona et al., 2024; van Wouwe et al., 2018) with 3,108 participants were included, for multi-arm trials, only one eligible intervention arm was selected to avoid double-counting participants. Heterogeneity test showed *I*^2^ = 75%, *p* < 0.0001; a random-effects model was used. Overall academic achievement was significantly higher in the exercise group (SMD = 0.17, 95% CI [0.01, 0.34], *p* = 0.04). This effect size is very small (trivial) according to Cohen’s conventional criteria (*d* < 0.2), indicating limited practical significance (see Figure 4).

##### 3.4.2.2 Effect on mathematics performance

**Figure 5:**
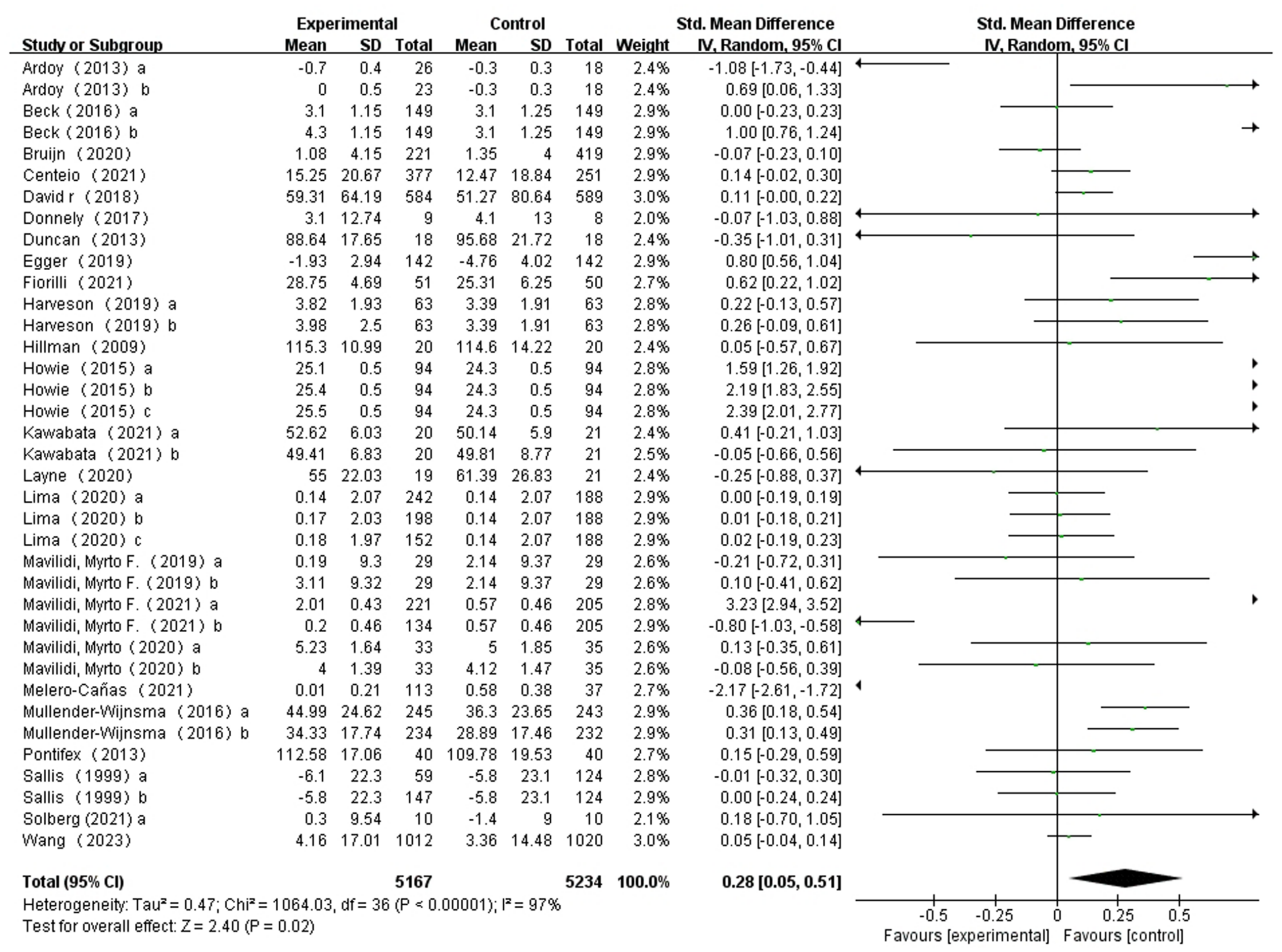
Forest plot of the effect of physical exercise on students’ mathematics performance. Standardized mean differences (SMD) and 95% confidence intervals (CI) are displayed for individual studies and the pooled estimate.

Twenty-three studies (Ardoy et al., 2013; Beck et al., 2016; Centeio et al., 2021; De Bruijn et al., 2020; Donnelly et al., 2017; Duncan and Johnson, 2013; Fiorilli et al., 2021; Harveson et al., 2019; Hillman et al., 2009; Howie et al., 2015; Kawabata et al., 2021; Layne et al., 2020; Lima et al., 2020; Lubans et al., 2018; Mavilidi et al., 2020; Mavilidi et al., 2019; Mavilidi and Vazou, 2021; Melero-Cañas et al., 2021; Mullender-Wijnsma et al., 2016; Parmenter et al., 2019; Pontifex et al., 2013; Sallis et al., 1999; Solberg et al., 2021a,b; Wang et al., 2023) with 10,401 participants were included. Heterogeneity test showed *I*^2^ = 97%, *p* < 0.00001, indicating substantial between-study variability. A random-effects model was used, consistent with our pre-specified approach for high heterogeneity. Mathematics performance was significantly higher in the exercise group (SMD = 0.28, 95% CI [0.05, 0.51], *p* = 0.02) (see Figure 5).

##### 3.4.2.3 Effect on spelling performance

Six studies (De Bruijn et al., 2020; Donnelly et al., 2017; Duncan and Johnson, 2013; Hillman et al., 2009; Mullender-Wijnsma et al., 2016; Pontifex et al., 2013) with 1,270 participants were included. Heterogeneity test showed *I*^2^ = 36%, *p* = 0.17; a fixed-effects model was used. Spelling performance was significantly higher in the exercise group (SMD = 0.13, 95% CI [0.02, 0.24], *p* = 0.02). This effect size is very small (trivial) based on Cohen’s criteria (*d* < 0.2), suggesting minimal practical significance.

##### 3.4.2.4 Effect on reading performance

Ten studies (Centeio et al., 2021; De Bruijn et al., 2020; Donnelly et al., 2017; Duncan and Johnson, 2013; Hillman et al., 2009; Mullender-Wijnsma et al., 2016; Parmenter et al., 2019; Pontifex et al., 2013; Sallis et al., 1999; Solberg et al., 2021a,b) with 2,677 participants were included. Heterogeneity test showed *I*^2^ = 67%, *p* = 0.0005; a random-effects model was used. No significant difference was found (SMD = 0.13, 95% CI [−0.03, 0.30], *p* = 0.10).

##### 3.4.2.5 Effect on language performance

Four studies (Ardoy et al., 2013; Duncan and Johnson, 2013; Melero-Cañas et al., 2021; Sallis et al., 1999)with 725 participants were included. Heterogeneity test showed *I*^2^ = 94%, *p* < 0.00001, indicating extreme between-study heterogeneity. A random-effects model was employed as pre-specified. No significant difference was found (SMD = 0.13, 95% CI [−0.57, 0.83], *p* = 0.72).

##### 3.4.2.6 Effect on cognitive inhibition

**Figure 6:**
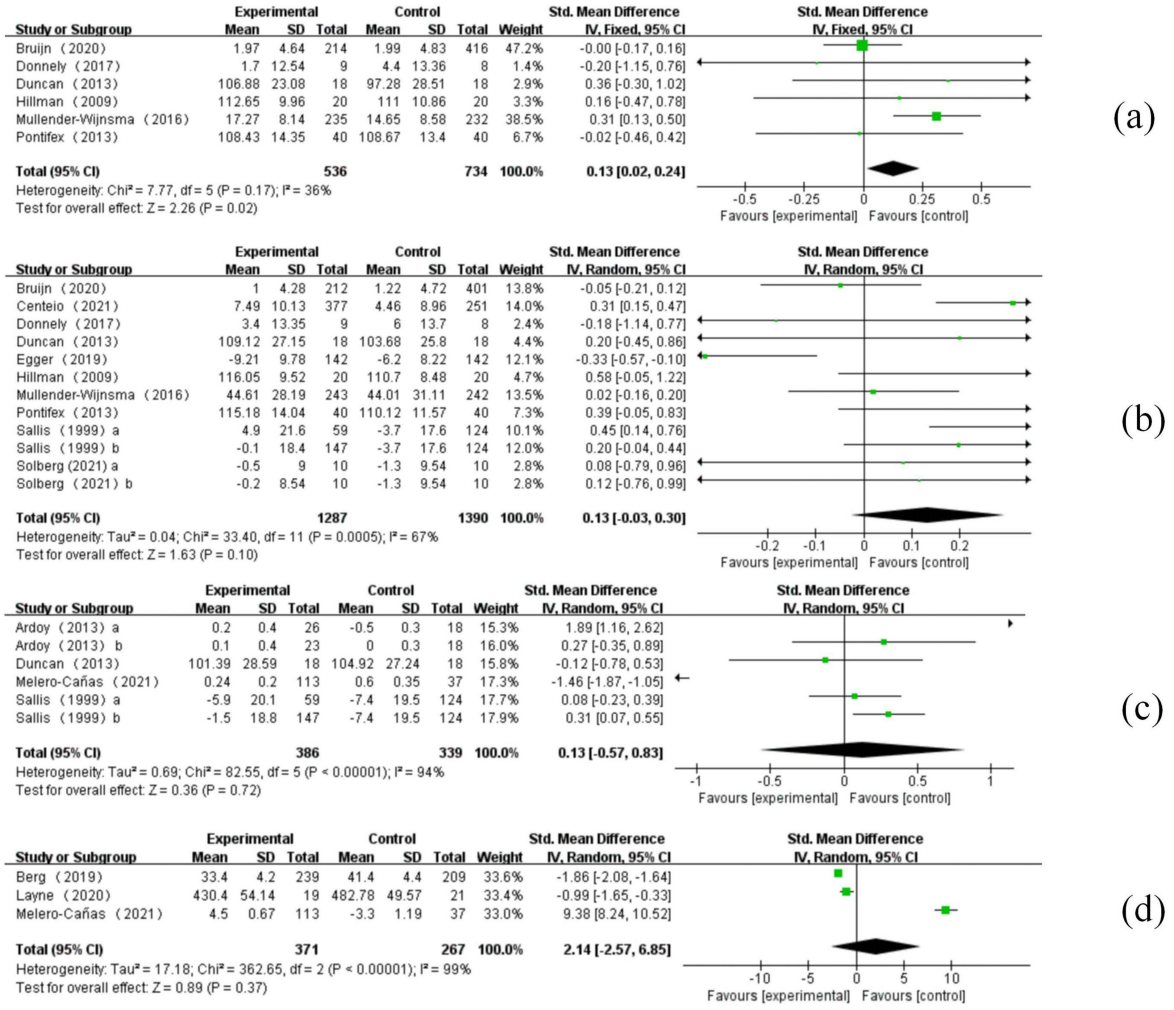
Forest plot illustrating the impact of physical exercise on students’ academic achievement. **(a)** Spelling performance **(b)** Reading performance **(c)** Language performance **(d)** Cognitive inhibition.

Three studies (Layne et al., 2020; Melero-Cañas et al., 2021; van den Berg et al., 2019) with 638 participants were included. Heterogeneity test showed *I*^2^ = 99%, *p* < 0.00001, reflecting almost all variability between studies. A random-effects model was used in line with our protocol. No significant difference was found (SMD = 2.14, 95% CI [−2.57, 6.85], *p* = 0.37). This very large effect size (SMD = 2.14) is likely driven by extreme outliers and high heterogeneity, so it should be interpreted with caution (see Figure 6).

### 3.5 Subgroup analyses

Subgroup analyses were performed for overall academic achievement and mathematics performance because of extremely high overall heterogeneity; the between-subgroup *I*^2^ = 66 and 74.9%, respectively. No subgroup analyses were conducted for emotional burnout, cynicism, exhaustion, professional efficacy, reading, language, or cognitive inhibition due to non-significant effects or low heterogeneity. For spelling performance, although the overall heterogeneity was low (*I*^2^ = 36%), a subgroup analysis by exercise type was conducted to explore potential differences in intervention efficacy. Subgroup analyses were conducted according to intervention type and student grade level.

#### 3.5.1 Subgroup analysis by intervention type for overall burnout

Subgroup analysis was performed for mindfulness and aerobic exercise. The overall result showed that exercise significantly reduced overall burnout (SMD = −0.33, 95% CI [−0.63, −0.04], *p* = 0.03), with moderate-to-high heterogeneity (*I*^2^ = 62%, *p* = 0.03).

**Figure 7:**
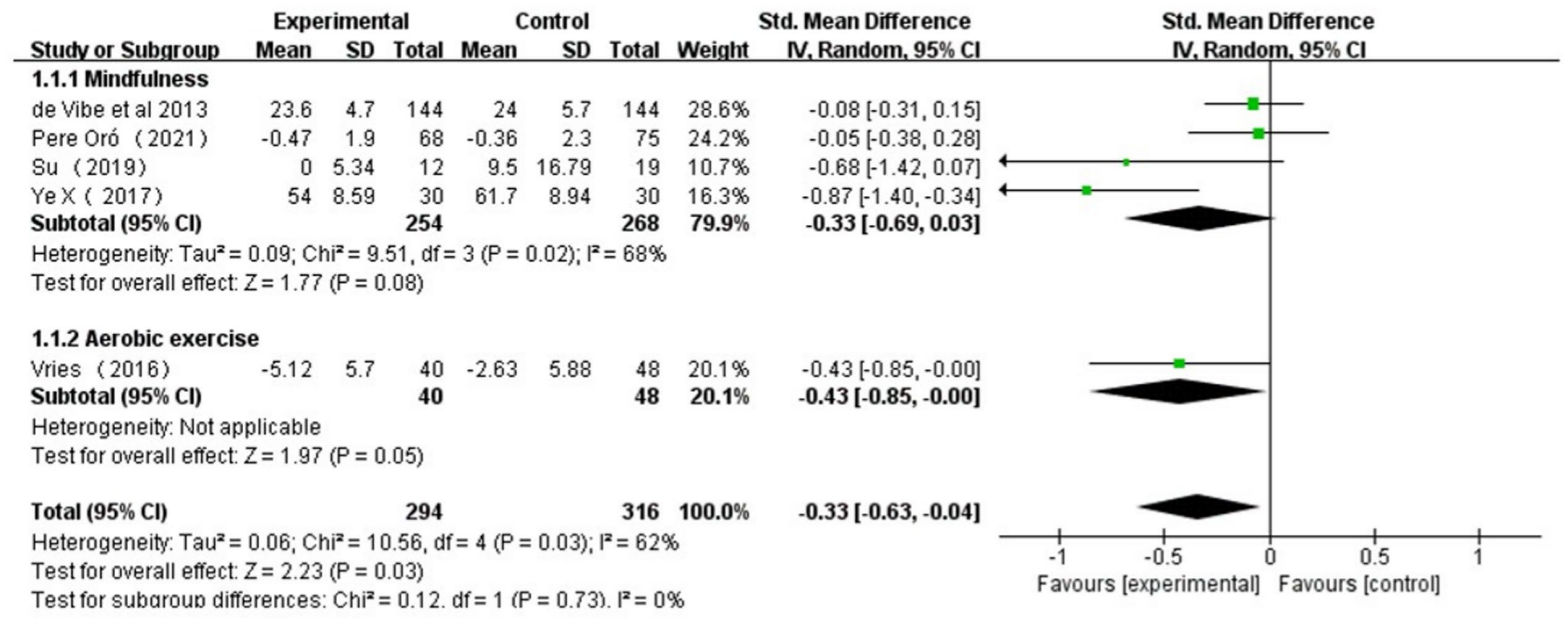
Subgroup analysis by intervention type (mindfulness vs. aerobic exercise) for overall academic burnout. Standardized mean differences (SMD) and 95% confidence intervals (CI) are presented for each subgroup and the between-subgroup comparison.

Mindfulness (4 studies) showed no significant effect (SMD = −0.33, 95% CI [−0.63, 0.03], *p* = 0.08). Aerobic exercise (1 study) showed marginal significance (SMD = −0.43, 95% CI [−0.85, 0.00], *p* = 0.05). No significant between-subgroup difference was found (*χ*^2^ = 0.12, d*f* = 1, *p* = 0.73, *I*^2^ = 0%) (see Figure 7).

#### 3.5.2 Subgroup analysis by grade level for overall academic achievement

Subgroup analysis was conducted by grade level (primary, junior high, senior high). The overall result showed that physical exercise significantly improved overall academic achievement (SMD = 0.17, 95% CI [0.01, 0.34], *p* = 0.04), with extremely high heterogeneity (*I*^2^ = 75%, *p* < 0.0001).

Significant differences were found across grades: Primary school: significant improvement (SMD = 0.40, 95% CI [0.10, 0.71], *p* = 0.009); Junior high school: no significant effect (SMD = −0.05, *p* = 0.80); Senior high school: no significant effect (SMD = 0.01, *p* = 0.86).

**Figure 8:**
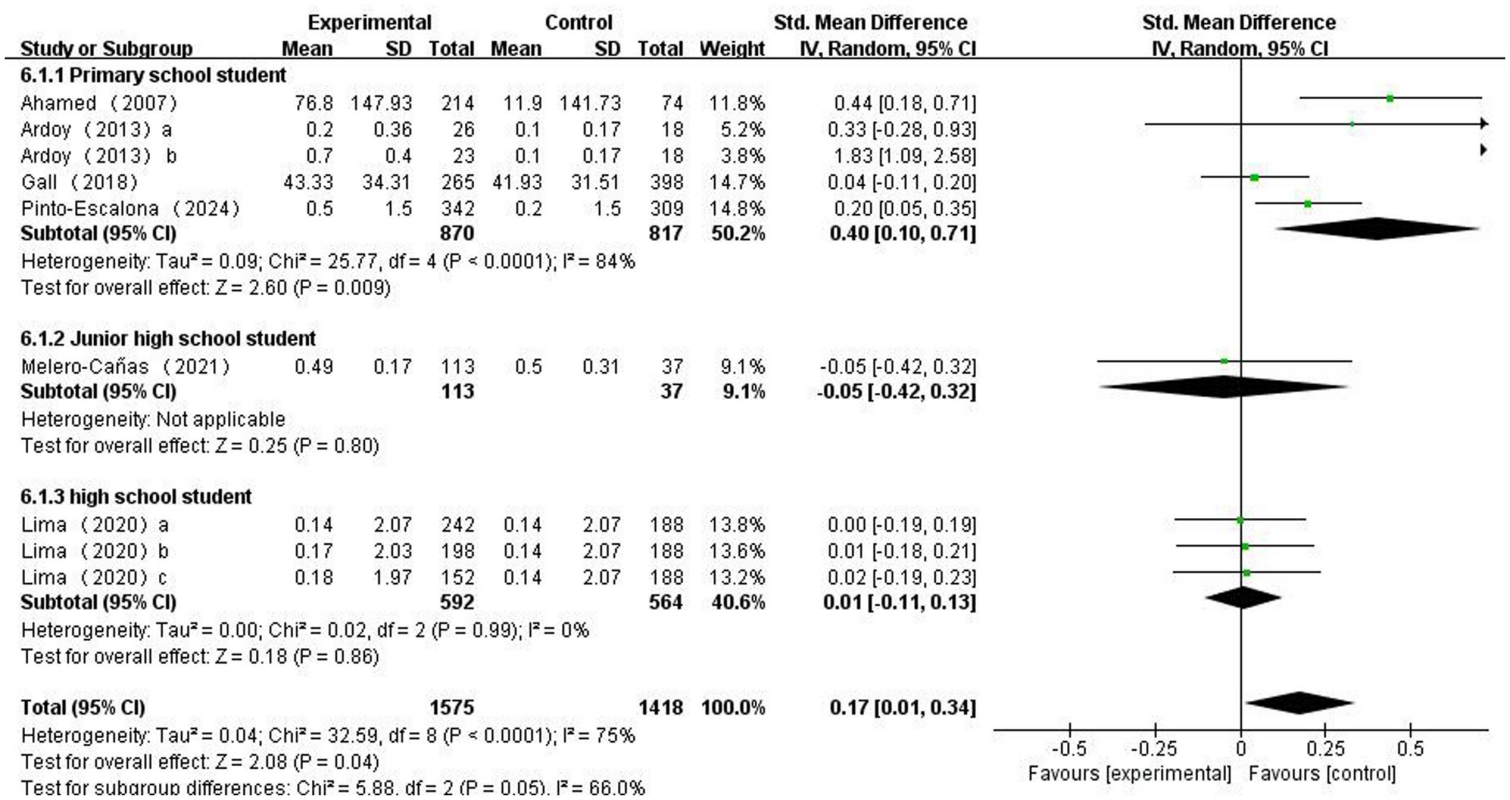
Subgroup analysis of overall academic achievement by student grade level (primary, junior high, senior high). Standardized mean differences (SMD) and 95% confidence intervals (CI) are reported for each subgroup and the between-subgroup test.

Between-subgroup difference was significant (*χ*^2^ = 5.88, d*f* = 2, *p* = 0.05, *I*^2^ = 66.0%). Grade level was a significant moderator. The beneficial effect of physical exercise on overall academic achievement existed only in primary school students (see Figure 8).

#### 3.5.3 Subgroup analysis by grade level for mathematics performance

The overall meta-analysis showed that physical exercise significantly improved mathematics performance (SMD = 0.28, 95% CI [0.05, 0.51], *p* = 0.02), with extremely high heterogeneity (*I*^2^ = 97%, *p* < 0.00001).

After subgrouping: Primary school: significant improvement (SMD = 0.47, 95% CI [0.15, 0.78], *p* = 0.004); Junior high school: no significant effect (SMD = −0.26, *p* = 0.41); Senior high school: no significant effect (SMD = 0.02, *p* = 0.70).

**Figure 9:**
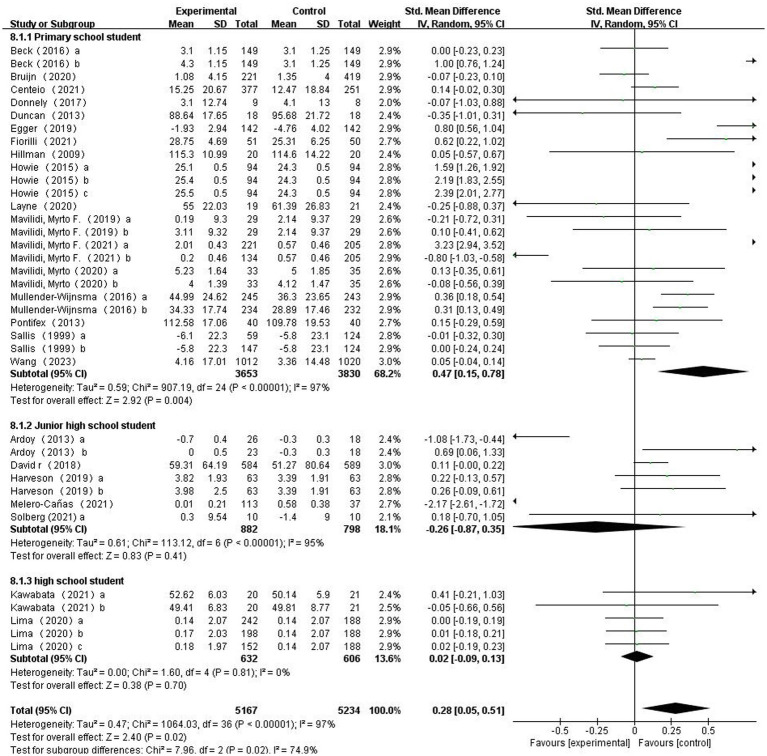
Subgroup analysis of mathematics performance by student grade level (primary, junior high, senior high). Standardized mean differences (SMD) and 95% confidence intervals (CI) are provided for each subgroup and the between-subgroup comparison.

Between-subgroup difference was significant (*χ*^2^ = 7.96, d*f* = 2, *p* = 0.02, *I*^2^ = 74.9%). The benefit only existed in primary school students (see Figure 9).

#### 3.5.4 Subgroup analysis by exercise type for spelling performance

The overall result showed that exercise significantly improved spelling performance (SMD = 0.13, 95% CI [0.02, 0.24], *p* = 0.02), with mild heterogeneity (*I*^2^ = 36%, *p* = 0.17).

**Figure 10:**
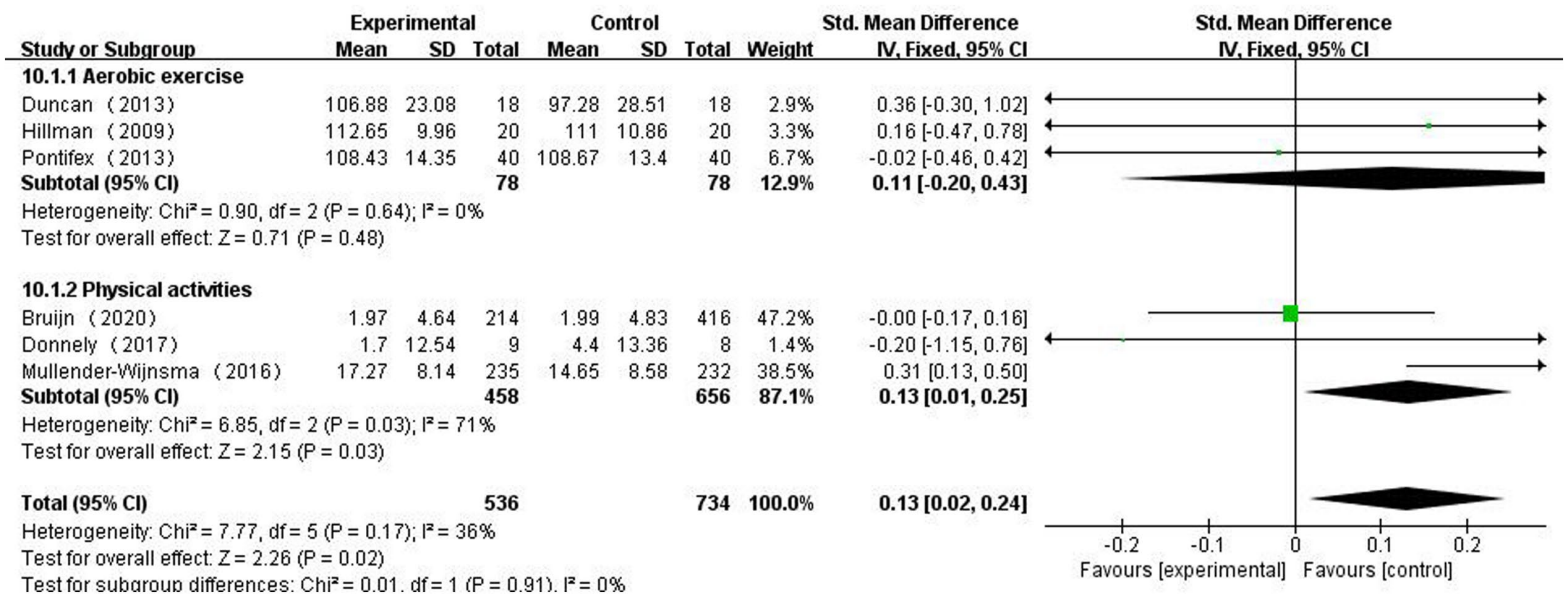
Subgroup analysis of spelling performance by exercise type (aerobic exercise vs. physical activity). Standardized mean differences (SMD) and 95% confidence intervals (CI) are shown for each subgroup and the between-subgroup difference.

Subgroup results: aerobic exercise showed no significant effect, while physical activity showed a significant benefit. No significant between-group difference was found (see Figure 10).

### 3.6 Sensitivity analysis

**Figure 11:**
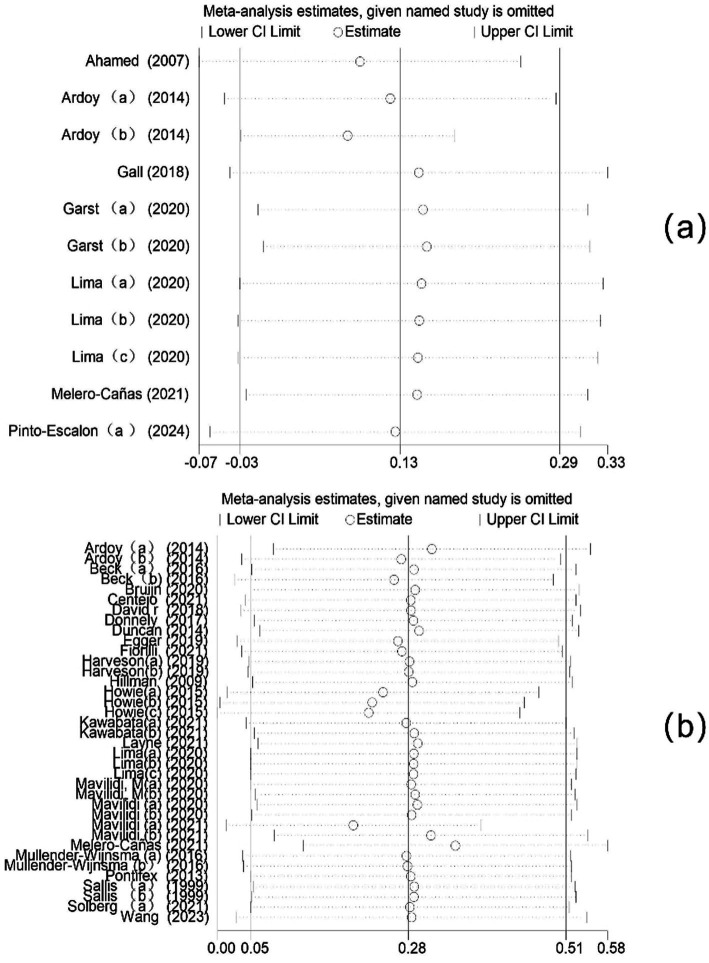
Sensitivity analysis of the impact of physical exercise on students’ overall academic achievement and mathematics performance; **(a)** academic burnout, **(b)** academic achievement.

One-study removed sensitivity analysis was conducted for overall academic burnout, overall academic achievement, mathematics performance, and spelling performance. After omitting any single study, the pooled results remained statistically significant and consistent, indicating robust findings (see Figure 11).

### 3.7 Publication bias assessment

**Figure 12:**
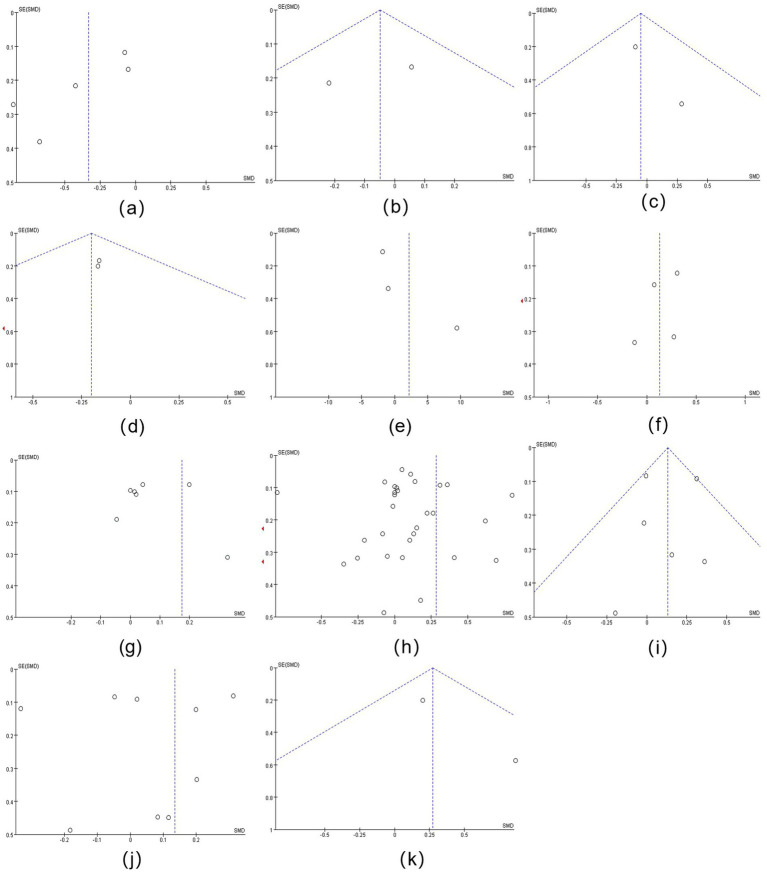
Detection of publication bias risk in the impact of physical exercise on student academic burnout and academic achievement; **(a)** General burnout **(b)** Emotional burnout **(c)** Cynicism **(d)** Exhaustion **(e)** Occupational efficacy **(f)** Overall academic achievement **(g)** Mathematics performance **(h)** Spelling performance **(i)** Reading performance **(j)** Language performance **(k)** Cognitive inhibition.

Publication bias was assessed for all outcomes. Funnel plots were visually inspected for all outcomes; quantitative Egger’s test was only performed for outcomes with 10 or more included studies (overall academic achievement and mathematics performance) (see Figure 12).

Egger’s regression was used for outcomes with ≥10 studies: Overall academic achievement: *t* = 0.80, *p* = 0.442; Mathematics performance: *t* = 0.82, *p* = 0.418, Both *p* > 0.05, indicating no significant publication bias.

## 4 Discussion

This review systematically evaluated the effectiveness of physical exercise on academic burnout and academic achievement among students. A total of 35 studies were included. The overall results showed that physical exercise reduced academic burnout and was associated with small but statistically significant improvements in academic achievement among students, especially for overall academic burnout, mathematics performance, and spelling performance. However, the evidence for improvements in emotional burnout, exhaustion, professional efficacy, cynicism, reading, cognitive inhibition, and language performance remain inconclusive. We also established a clear definition of physical exercise and restricted mindfulness to mind–body integrated interventions to avoid conceptual ambiguity. Consistent with prior research, physical exercise significantly reduced overall academic burnout, while other burnout dimensions showed non-significant trends. This may be related to short intervention duration and low-intensity physical exercise.

Regarding academic achievement, the results of this study are generally consistent with previous findings: various forms of physical exercise [aerobic exercise (Harveson et al., 2019; Mavilidi et al., 2020; Pontifex et al., 2013), physical games (Melero-Cañas et al., 2021), physical education (Sallis et al., 1999)] can reduce overall academic burnout and alleviate academic anxiety, and thereby improve overall academic achievement, mathematics performance, and spelling performance (LeBouthillier and Asmundson, 2017). Published meta-analyses have shown that mathematics instruction integrated with physical activity significantly improves mathematics scores (Mavilidi and Vazou, 2021)and promotes students’ cognitive, affective, and motivational development (Li et al., 2023; Norris et al., 2020; Wang et al., 2023), which supports the present findings. Further subgroup analysis revealed that physical exercise improved mathematics performance more significantly in primary school students. This age-related benefit may be linked to developmental differences in cognitive responsiveness to physical activity. Meta-analysis indicated that the intervention group performed significantly better than the control group in mathematics performance, overall academic achievement, and spelling performance, all reaching statistical significance. These results suggest that physical exercise enhances academic achievement by stimulating learning interest and relieving academic anxiety (Syväoja et al., 2024). In contrast, no significant improvements were observed in reading or language performance (Resaland et al., 2016), consistent with some previous studies (Wang et al., 2023). This may be attributed to disciplinary characteristics: improvements in reading and language rely more on long-term accumulation, making it difficult for short-term physical exercise to produce detectable effects. Notably, the included interventions varied substantially in type (aerobic exercise, karate, physical activity breaks, games), duration (2 weeks to 24 months), frequency (1–8 sessions/week), and intensity (low to high). Due to incomplete and inconsistent reporting of dosage parameters across studies, formal moderator analyses of dose–response relationships were not feasible in the current meta-analysis. Instead, we comprehensively discussed these potential moderating factors and their plausible influences on effect sizes in the present Discussion section.

Although the overall trend indicated that physical exercise exerted positive effects on students’ academic burnout and academic achievement, effect sizes and significance levels for overall academic burnout and mathematics performance differed from some studies, suggesting that the magnitude of academic burnout reduction varies substantially across different exercise intervention protocols (Bull et al., 2020; Chaput et al., 2020). This study found significant improvements in mathematics performance following physical exercise (Beck et al., 2016; Mullender-Wijnsma et al., 2016; Solberg et al., 2021a,b), but effect sizes differed from studies using only aerobic exercise (Harveson et al., 2019; Mavilidi et al., 2020; Pontifex et al., 2013; Solberg et al., 2021a,b). Evidence indicates that not all physical interventions equally promote academic achievement (Bull et al., 2020; Chaput et al., 2020). Further analysis revealed that interventions combining exercise with academic content or involving high cognitive challenge (van den Berg et al., 2019) produced greater improvements in mathematical logic and spelling ability than simple running or cycling. Some previous comparative studies used only simple physical activities, which lacked engagement of executive functions (Pinto-Escalona et al., 2024), resulting in weaker academic benefits than those observed in the present study. The low-intensity physical activities reported in the included studies (including various types) emphasized coordinated physical and mental engagement, leading to stronger effects on mathematics and spelling performance than on overall academic achievement (Mavilidi et al., 2018; Parmenter et al., 2019). Subgroup analysis showed that the effect size of physical exercise on spelling performance was significantly larger in primary school students than in older groups. This is mainly attributed to the high sensitivity of executive function development during primary school.

Existing meta-analyses on academic burnout (Ahamed et al., 2007; Fisher et al., 2016; Kewei et al., 2019; O’Driscoll et al., 2019; Oró et al., 2021; Xin et al., 2017) have focused on mindfulness or psychological interventions. In contrast, this study focused exclusively on physical exercise, supplementing evidence in the field of academic burnout intervention. Notably, many mindfulness studies (Kewei et al., 2019; O’Driscoll et al., 2019; Oró et al., 2021; Xin et al., 2017) focused on highly stressed medical or university students, whereas the present sample covered students aged 6 to 24 years. Comparative analysis indicated that combined physical activity and mindfulness produced more positive effects in this broad student population (Alsaraireh and Aloush, 2017; Sala et al., 2021; Yan et al., 2023). Moreover, this study included a large sample of Chinese students with high academic burnout (Kewei et al., 2019; Xin et al., 2017). Chinese students generally experience heavier academic pressure and higher baseline burnout (Xin et al., 2017), so physical exercise and mindfulness interventions showed stronger beneficial effects (Dahlin and Runeson, 2007; Sekhar et al., 2021; Zieff et al., 2022). Some Western studies (Ahamed et al., 2007) emphasized that physical exercise does not interfere with learning, rather than reducing severe burnout. Differences in research purpose and baseline levels contributed to divergent effect sizes.

This study has several methodological strengths. We searched three electronic databases with no language restrictions. This strategy minimized publication bias and ensured the breadth and representativeness of included studies, improving generalizability. Dual independent literature screening, data extraction, and risk of bias assessment were performed, with a clear arbitration mechanism for disagreements. This procedure effectively reduced subjective bias and ensured data accuracy and reliability. Finally, we systematically conducted heterogeneity testing, sensitivity analysis, and publication bias assessment, comprehensively evaluating the stability and robustness of results, making conclusions more interpretable and convincing rather than simple data pooling. Despite these strengths, this study has limitations. Despite strict screening, some included studies had methodological limitations, such as lack of blinding and missing attrition data. Furthermore, despite our detailed subgroup analyses, high heterogeneity still persisted across several key outcomes. This was mainly driven by unstandardized intervention protocols, inconsistent reporting of exercise intensity, diverse assessment tools, and the wide age range of participants (6–24 years). Additionally, the included literature was predominantly published in Western countries, with a limited number of Chinese studies, and a small portion of references were relatively outdated. Such uneven regional distribution and partial outdated citations may restrict the generalizability of the findings. The incomplete documentation of exercise dosage and intensity in original studies also limited further exploration of heterogeneity sources, which should be addressed in future well-designed research. These potential risks of bias may have influenced the results to some extent. Furthermore, to address the conceptual ambiguity of physical exercise, we established a clear and consistent definition of physical exercise and included mindfulness practices only when they were integrated with physical exercise (i.e., mind–body exercise), which effectively reduced heterogeneity caused by inconsistent intervention definitions.

Although Egger’s test and sensitivity analysis verified the robustness of the results for the main outcomes, moderate-to-high heterogeneity remained for some outcomes, which may still affect result stability despite corrective measures. Notably, considerable between-study heterogeneity was observed in the analyses of mathematics performance (*I*^2^ = 97%) and overall academic achievement (*I*^2^ = 75%). To clarify the potential sources of such variability, we conducted in-depth qualitative interpretation combined with quantitative meta-regression, which revealed that the substantial heterogeneity was jointly driven by multiple interrelated factors. Specifically, the included interventions spanned a broad range of forms, including aerobic exercise, mindfulness practice, karate training, physical activity games, and academically integrated physical activity, and interventions involving higher cognitive engagement generally generated larger effect sizes than basic physical exercise, thereby contributing to marked variation in intervention effects. Meanwhile, the significant moderating role of grade level indicated that the academic benefits of physical exercise were predominantly evident among primary school students but not in junior or senior high school populations, introducing substantial age-related variability. Furthermore, intervention dosage was highly inconsistent across studies, with large variations in session duration, weekly frequency, and total intervention period, and the reporting of exercise intensity was incomplete or unstandardized, both of which amplified heterogeneity. In addition, outcome assessment relied on diverse tools including standardized academic tests, school-based examinations, and teacher ratings, which differed in measurement sensitivity, scoring rubrics, and normative references. Although cultural variations in educational systems, academic pressure, and school physical education policies may also contribute to cross-study differences, such effects were not statistically supported by the meta-regression model. Collectively, our meta-regression analyses quantitatively confirmed grade level and intervention type as significant moderators, offering a rigorous and comprehensive explanation for the high heterogeneity detected in the current meta-analysis. Therefore, future studies should conduct large-sample, multicenter, rigorously designed RCTs with long-term follow-up. We recommend conducting studies in natural school settings to improve ecological validity, with detailed documentation of interventions, implementation procedures, and outcome measures to reduce bias and provide higher-quality evidence. Finally, future research should use refined designs to systematically examine how factors such as age group, baseline academic pressure, and sociocultural context moderate intervention effects.

## 5 Conclusion

This study indicates that physical exercise may exert positive effects on academic burnout and academic achievement among students, especially in reducing overall academic burnout. Although statistically significant improvements were observed in overall academic achievement and spelling performance, the effect sizes were very small and of limited practical significance. Sensitivity analysis and publication bias assessment indicated that the present findings were robust, with no significant publication bias. Furthermore, subgroup analyses based on various variables further verified the robustness of the results and enhanced the credibility of the conclusions. Notably, extremely high heterogeneity was observed for mathematics, language performance, and cognitive inhibition, which reflects substantial variability in intervention protocols and outcome measures across included studies. This study addresses gaps in the existing literature by focusing exclusively on RCTs that integrate mindfulness and physical exercise, and systematically examining academic burnout alongside academic achievement, which distinguishes it from prior meta-analyses focused only on physical activity and academic outcomes, resolves inconsistencies among existing studies, and improves the theoretical system related to physical exercise and student academic outcomes. It also provides evidence-based implications for schools, families, and educational authorities to develop physical exercise interventions aimed at alleviating academic burnout and improving academic achievement. Nevertheless, several limitations exist: unbalanced sample sizes and geographic distributions, relatively high risk of bias in some included studies, and inevitable heterogeneity was observed across intervention protocols (e.g., exercise type, duration), though the overall pooled results remained robust, which may limit the generalizability of the findings. Future studies should conduct more high-quality, large-sample, and long-term follow-up RCTs, standardize exercise intervention protocols and outcome measurement tools, and expand geographic coverage to improve the generalizability of conclusions.

## Data Availability

The original contributions presented in the study are included in the article/Supplementary material, further inquiries can be directed to the corresponding author.
